# Large Pericardial Cyst Presenting as Acute Cough: A Rare Case Report

**DOI:** 10.1155/2018/4796903

**Published:** 2018-12-05

**Authors:** Michael Makar, Gabriel Makar, Kerolos Yousef

**Affiliations:** ^1^Geisinger Commonwealth School of Medicine, 525 Pine Street, Scranton, PA 18510, USA; ^2^Cooper Medical School of Rowan University, 401 Broadway, Camden, NJ 08103, USA; ^3^Department of Anesthesiology, Geisinger Medical Center, 100 N. Academy Ave., Danville, PA, USA

## Abstract

Pericardial cysts are an uncommon cause of mediastinal masses and may be found incidentally on imaging. Symptoms commonly include cough, chest pain, and shortness of breath elucidating a broad differential on examination. Diagnosis is predominantly made using imaging modalities, such as CT, MRI, and CXR with treatment including resection for symptomatic cysts and observation for asymptomatic cysts. Due to a lack of specific signs and symptoms towards identifying pericardial cysts, many are identified at a later stage requiring resection by video-assisted thoracoscopic surgery (VATS). We present the rare case of a patient presenting with a sudden onset cough, shortness of breath, and pleuritic chest pain found to have a large pericardial cyst.

## 1. Introduction

Pericardial cysts are uncommon benign congenital anomalies located in the mediastinum. They typically present in the middle mediastinum, representing approximately 33% of mediastinal cysts and 6–7% of mediastinum masses [1]. Pericardial cysts are increasingly rare with a majority detected incidentally and an incidence rate of 1 in 100,000 [2–4]. Most cysts are identified in the third or fourth decade of life equally affecting both males and females [2]. Pericardial cysts are commonly congenital and largely asymptomatic; however, symptoms may present in light of complications such as mass effect, rupture, inflammation, and hemorrhage [5, 6]. Other documented etiologies of pericardial cysts include inflammatory etiologies such as rheumatic pericarditis, bacterial infection particular from tuberculosis, trauma, and postcardiac surgery [5]. The management of patients with pericardial cysts includes conservative management with routine follow-up, percutaneous aspiration, and surgery. Typically, a cyst will only require follow-up for observation for increase in size; however, an enlarging or symptomatic cyst may require surgical removal [5]. We present the case of a patient with acute onset worsening cough, shortness of breath, and sudden onset pleuritic chest pain found to have a large pericardial cyst.

## 2. Case Presentation

**Figure 1:**
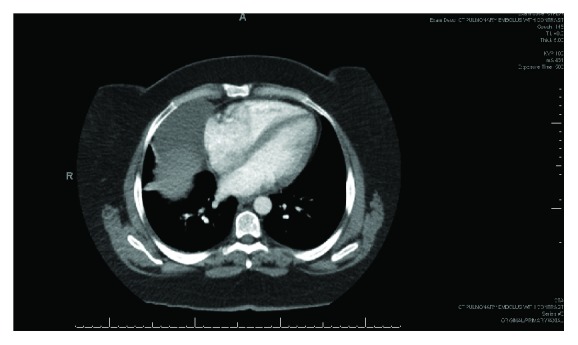
CT chest with contrast demonstrating a pericardial cyst.

**Figure 2:**
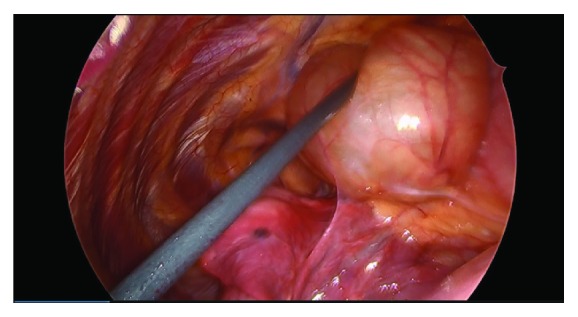
Intraoperative view of the pericardial cyst.

A 43-year-old female with a past medical history of severe allergy-induced asthma and chronic nasal drip presented to the clinic with worsening cough and chest tightness for 10 days and was treated for bronchitis with steroids and antibiotics. She then presented to the emergency department with severe and acutely worsening shortness of breath, sudden onset pleuritic chest pain, and sharp radiating pain between both shoulder blades with deep inspiration. At time of presentation, the patient was afebrile with vital signs within the normal limits. Lab work was insignificant with negative leukocyte count and negative cardiac enzymes. A chest X-ray demonstrated an abnormal right cardiomediastinal silhouette with large opacity over the right mediastinum adjacent to the right atrial border. A follow-up CT scan revealed a large right-sided mass adjacent to the right atrium and extending into the right chest measuring 5.1 cm × 9 cm × 4.3 cm (Figure 1). Her last imaging study was a fluoroscopy study 10 years ago that showed no indications for a mediastinal mass. An echocardiogram revealed a normal ejection fraction (55–59%), no wall motion abnormalities, and a cyst near the right atrium. The patient had continued pleuritic chest pain and difficulty breathing and the decision was made to perform video-assisted thoracoscopic surgery (VATS) for pericardial cyst removal. The patient underwent general anesthesia with a 37 French left-sided double-lumen tube. Standard ASA monitors were applied. Two large-bore peripheral IVs and an arterial line were placed for continuous blood pressure monitoring. Intraoperatively, a large cystic lesion was adherent to the pericardium (Figure 2). There was no solid component and no obvious communication with the pericardium. Part of the cyst wall was left on the phrenic nerve to preserve it. The patient tolerated the procedure well, had no postoperative complications, and was discharged home on postoperative day number two. The final pathology report revealed benign, acute inflammatory pericardial cyst.

## 3. Discussion

Pericardial cysts are rare, benign causes of mediastinal masses that are largely asymptomatic in nature. With a low incidence rate and most cases presenting incidentally, pericardial cysts are difficult to identify without the use of imaging modalities. Symptomatic pericardial cysts are rare and are a result of various etiologies ranging from inflammatory pathology to mass effect [5].

This case is a unique presentation of a patient who presented with acute onset worsening shortness of breath, cough, and sudden onset pleuritic chest pain. It is unclear when the cyst began to develop and how rapidly it developed prior to her presentation. However, imaging 10 years prior revealed no evidence of a cyst. Her recent episode of bronchitis that was treated with steroids and antibiotics may have triggered a rapid enlargement of the cyst, precipitating her symptoms that resulted in her admission to the hospital. Her presentation of pleuritic chest pain and dyspnea are common symptoms for many diagnoses including pulmonary embolism, pneumonia, pneumothorax, and pericarditis. In addition, pericardial cysts may be confused with other pathologies, and the initial read on CXR was read as Morgagni's hernia. Although rare, it is important for clinicians to include a pericardial cyst in the differential diagnosis of patients who present with pleuritic chest pain and a mass on CT imaging.

Cysts range in size from 2 to 3 cm but have been reported as large as 28 cm [7]. The cysts appear as well-defined or oval masses in contact with the heart. Most frequently, the cyst is found in the right costophrenic angle but may also be found elsewhere in the mediastinum [4, 8]. On a CT scan, a pericardial cyst typically appears as a well-defined, nonenhanced homogenous oval mass adjacent to the pericardium [9]. If symptoms are present, they are usually due to compression of adjacent organs, rupture, or inflammation and include atypical chest pain, dyspnea, and persistent cough [4]. A cyst located adjacent to the pericardium may lead to symptoms due to medullated fibers that respond to stretching or touching of the pericardium [10]. Cardiac tamponade, infection, hemorrhage, and sudden death are life-threatening complications that have been previously reported in these patients [4]. Management of a pericardial cyst depends on the size of the cyst and patient presentation. Observation, percutaneous drainage, and resection are three possible options. Observation is recommended unless the patient has chest pain, tamponade, or there is uncertainty of malignant potential. Serial transthoracic echocardiography is used to monitor asymptomatic patients and ensure a benign course in which the pericardial cyst can resolve spontaneously [9] In addition, transesophageal echocardiography (TEE) is also used to help identify the cyst in atypical locations and differentiate it from other pathologies such as an aneurysm, appendage, or solid tumors. Antonini-Canterin et al. presented two case reports in which the TEE was used to identify the cyst in two atypical locations. One cyst appeared in the posterior wall of the right pulmonary artery and another cyst was found compressing the left atrium and the outflow of the pulmonary veins [11]. Furthermore, cardiac computed tomographic scanning can be used alongside the TEE to help identify the relationship between the cyst and the pericardium [11]. Resection with either a thoracotomy or video-assisted thoracoscopic surgery (VATS) has been the preferred approach for patients with a symptomatic cyst [5]. Surgery has been identified as the only definitive cure. Since operative risks of minimally invasive techniques are extremely low, it would seem reasonable to offer resection for all pericardial cysts in otherwise healthy patients for whom the risk of surgery is low [5].

## 4. Conclusion

Although rare, pericardial cysts can present with various clinical presentations and can range from asymptomatic to life-threatening in nature. This case is a unique presentation of a patient who presented with the development of a pericardial cyst with rapidly worsening symptoms that required resection with VATS. VATS has numerous advantages including decreased pain and shorter postoperative recovery and has become a common treatment option for patients with enlarging or symptomatic cysts. Physicians must not neglect to include pericardial cysts into the broad differential of patients presenting with acute onset cough, shortness of breath, or pleuritic chest pain.
